# Racial and ethnic disparities in HIV diagnoses among heterosexually active persons in the United States nationally and by state, 2018

**DOI:** 10.1371/journal.pone.0257583

**Published:** 2021-09-20

**Authors:** Erika G. Martin, Bahareh Ansari, Rachel Hart-Malloy, Dawn K. Smith, Kevin P. Delaney, Thomas L. Gift, Andrés A. Berruti, Monica Trigg, Eli S. Rosenberg

**Affiliations:** 1 Department of Public Administration and Policy, Rockefeller College of Public Affairs and Policy, University at Albany, Albany, New York, United States of America; 2 Center for Collaborative HIV Research in Practice and Policy, School of Public Health, University at Albany, Albany, New York, United States of America; 3 Department of Information Science, College of Emergency Preparedness, Homeland Security and Cybersecurity, University at Albany, Albany, New York, United States of America; 4 New York State Department of Health, Office of Sexual Health and Epidemiology, AIDS Institute, Albany, New York, United States of America; 5 Department of Epidemiology and Biostatistics, School of Public Health, University at Albany, Albany, New York, United States of America; 6 Division of HIV/AIDS Prevention, National Center for HIV, Viral Hepatitis, STD, and TB Prevention, Centers for Disease Control and Prevention, Atlanta, Georgia, United States of America; 7 Department of Epidemiology, Rollins School of Public Health, Emory University, Atlanta, Georgia, United States of America; City University of New York Graduate School of Public Health and Health Policy, UNITED STATES

## Abstract

**Background:**

Despite declining HIV infection rates, persistent racial and ethnic disparities remain. Appropriate calculations of diagnosis rates by HIV transmission category, race and ethnicity, and geography are needed to monitor progress towards reducing systematic disparities in health outcomes. We estimated the number of heterosexually active adults (HAAs) by sex and state to calculate appropriate HIV diagnosis rates and disparity measures within subnational regions.

**Methods:**

The analysis included all HIV diagnoses attributed to heterosexual transmission in 2018 in the United States, in 50 states and the District of Columbia. Logistic regression models estimated the probability of past-year heterosexual activity among adults in three national health surveys, by sex, age group, race and ethnicity, education category, and marital status. Model-based probabilities were applied to estimated counts of HAAs by state, which were synthesized through meta-analysis. HIV diagnoses were overlaid to calculate racial- and ethnic-specific rates, rate differences (RDs), and rate ratios (RRs) among HAAs by sex and state.

**Results:**

Nationally, HAA women have a two-fold higher HIV diagnosis rate than HAA men (rate per 100,000 HAAs, women: 6.57; men: 3.09). Compared to White non-Hispanic HAAs, Black HAAs have a 20-fold higher HIV diagnosis rate (RR, men: 21.28, women: 19.55; RD, men: 15.40, women: 31.78) and Hispanic HAAs have a 4-fold higher HIV diagnosis rate (RR, men: 4.68, RD, women: 4.15; RD, men: 2.79, RD, women: 5.39). Disparities were ubiquitous across regions, with >75% of states in each region having Black-to-White RR ≥10.

**Conclusion:**

The racial and ethnic disparities across regions suggests a system-wide failure particularly with respect to preventing HIV among Black and Hispanic women. Pervasive disparities emphasize the role for coordinated federal responses such as the current Ending the HIV Epidemic (EHE) initiative.

## Introduction

Although human immunodeficiency virus (HIV) diagnosis rates among heterosexually active adults (HAAs) in the United States (US) have declined, reducing racial and ethnic inequities in HIV prevention and treatment is a priority in national and jurisdictional HIV strategies [1–3]. A recent analysis among HAA men in the US from 2014–2018 revealed substantial racial and ethnic inequities using 12 separate disparity measures [4,5]. Although HIV diagnosis rates are highest among persons reporting male-to-male sexual contact, 9.4% of new diagnoses among males and 84.6% among females aged ≥13 years are heterosexually acquired [6]. To meet national, state, and local “ending the epidemic” goals, further reduction of new infections among HAAs is needed, particularly among women.

Numerous factors contribute to inequities in HIV acquisition and health outcomes. Social and structural factors include poverty, unstable housing, incarceration, socioeconomic status, educational attainment, access to quality HIV prevention and care, and racial discrimination [7]. A recent analysis of durable viral suppression among adolescents and young adults nationally found that while disparities existed for all racial and ethnic groups, Black persons had the lowest durable viral suppression (36.1%, compared to 50.8%, 46.7%, and 47.3% among persons identifying as White, Hispanic, or other groups), which in turn increases transmission risk [8]. There are several reasons why viral suppression is lower among Black populations. First, minority patients are less likely to have healthcare providers of the same racial and ethnic identity; congruence in identity is associated with better patient-provider relationships, and lower cultural competence among providers is associated with worse HIV care outcomes [9]. Second, lower health literacy among Black persons with HIV may also negatively impact adherence to antiretroviral therapy [10]. Furthermore, structural racism, discrimination, and mistrust in the health system create barriers to HIV services utilization among Black persons [11]. These mechanisms also apply to other racial and ethnic minority groups.

Appropriate calculations of diagnosis rates by HIV transmission category, race and ethnicity, and geography are needed as one important metric in the HIV care continuum to monitor progress towards reducing systematic disparities in health outcomes that are the result of unjust social, economic, and environmental conditions. Racial and ethnic disparities in HIV diagnoses among HAA men and women are documented nationally [4,5,12], but state-level estimates for men and women are lacking. Building on recent work estimating HIV diagnoses rates among men who have sex with men (MSM) [13,14], we use meta-analysis to estimate state-level populations of HAAs, HIV diagnosis rates by sex, and an absolute and relative disparity measure of Black-White and Hispanic-White disparities in diagnosis rates. We use the term HAA, rather than heterosexual, because sexual activity and sexual orientation differ. The term “heterosexual” denotes sexual orientation, which cannot be deduced from the data. Instead, “heterosexually active” is used to denote reported sexual activity with someone of the opposite sex.

## Materials and methods

### Analytic overview

**Fig 1:**
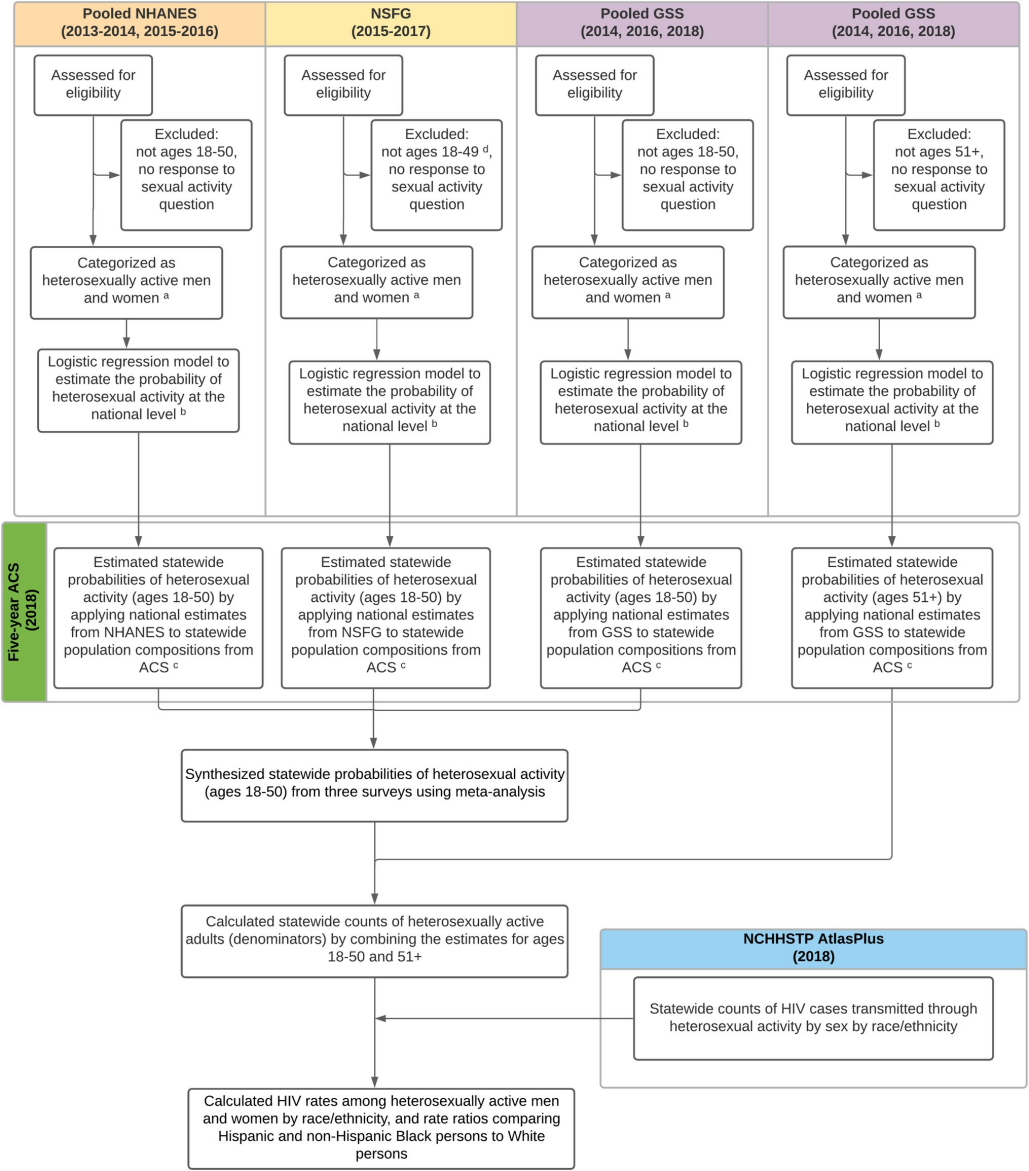
Analysis flow chart. Abbreviations: American Community Survey (ACS), General Social Survey (GSS), National Center for HIV/AIDS, Viral Hepatitis, STD, and TB Prevention (NCHHSTP), National Health and Nutrition Examination Survey (NHANES), National Survey of Family Growth (NSFG). ^a^ Heterosexual activity among men defined as men who have had sex with women exclusively in the past 12 months. Heterosexual activity among women defined as women who have had sex with men exclusively and women who have had sex with both men and women in the past 12 months. ^b^ The logistic regression model contains covariates for age group (18–29, 30–39, and 40–50), sex (female or male), race and ethnicity (White, Black, Hispanic, and other), education category (high school and lower, some college, and college graduate and above), and marital status (never married; married; and widowed, separated, or divorced). This yielded 216 unique demographic strata. The logistic regression model for GSS (ages 51+) did not contain a covariate for age, yielding 72 strata. ^c^ To estimate state-wide probabilities of heterosexual activity, we first calculated the national estimate of recent heterosexual activity for 216 strata among persons aged 18 to 50 (all combinations of sex, race and ethnicity, education, age, and marital status categories) and subsequently applied these estimates to statewide population compositions of these strata. We repeated that exercise for the 72 strata in the GSS-based model estimate for persons aged 51+. ^d^ NSFG covers respondents up to 49 years. There were a few participants that were 50 years of age.

Consistent with prior work to estimate populations of MSM [13,14] and HAAs [15] nationally, we used a four-step process to develop state-level estimated HIV diagnosis rates, rate differences (RDs), and rate ratios (RRs) of racial and ethnic disparities among HAAs (see Fig 1). First, we used logistic regression models to estimate the probability of past-year heterosexual activity among persons aged ≥18 years in three national health surveys by sex, age group, race and ethnicity, education category, and marital status. Second, model-based probabilities were applied to the American Community Survey (ACS) to generate proportions of HAA men and women by state. Third, we used meta-analysis to synthesize estimated probabilities across surveys; these were applied to the ACS to generate estimated populations of HAAs. Fourth, HIV diagnoses were overlaid to calculate state-level, sex-specific rates by race and ethnicity, and RDs and RRs as measures of racial and ethnic disparities.

### Data sources and methods for HAAs

Four publicly-available national surveys were used to estimate HAA populations: National Health and Nutrition Examination Survey (NHANES; pooled 2013–2014 and 2015–2016 waves) [16], National Survey of Family Growth (NSFG; 2015–2017 wave) [17], General Social Survey (GSS; pooled annual surveys from 2014, 2016, and 2018) [18], and ACS (5-year estimates from 2014–2018) [19]. Heterosexual activity among men was defined as self-reported sex with women exclusively in the past year. Heterosexual activity among women was defined as self-reported sex with men exclusively, or sex with both men and women in the past year. Women who had both male and female sex partners were included because while there are occasional case reports of female-to-female HIV transmission, this mode of transmission is rare [20] and thus we were inclusive of all women who reported male sexual partners. The “past year” definition was used to represent current sexual activity.

In the first stage, four logistic regression models estimated the probability of past-year heterosexual activity nationally. Three models developed these estimates separately for adults 18–50 years in NHANES, NSFG, and GSS; this age range was common to all surveys. Models included covariates and their two-way interactions for: age group (18–29, 30–39, and 40–50), sex (female or male), race and ethnicity (White non-Hispanic, Black non-Hispanic, Hispanic, and all other races combined), education category (high school and lower, some college, and college graduate and above), and marital status (never married; married; and widowed, separated, or divorced). The models were developed with an intent to improve their predictive power rather than limiting to the most significant coefficients from a stepwise procedure. The covariates and their categories were selected based on a review of demographic covariates used in prior literature [15], consistency across the three surveys, availability at the state level in the ACS (for the second stage described below), and ability to ensure at least 5 observations in each stratum for sufficient degrees of freedom. Regarding the categorization of race and ethnicity, all other races were combined due to the smaller numbers of survey participants in other race and ethnicity groups when stratifying by state, age group, race and ethnicity, marital status, etc. as described below, and will be referred to as “other races.” A fourth model estimated the probability of past-year heterosexual activity among adults aged ≥51 years in the GSS, using all covariates except age group because there were too few observations in the higher age group to generate a reliable estimate if the model included additional age group stratifications. All models used available survey weights. NHANES weights were divided by two because we pooled 2013–2014 and 2015–2016 waves. GSS survey weights were not altered because they adjust for non-response, not US population representativeness.

In the second stage, the ACS was used to project the percentage of state populations in the 216 strata (all age group, sex, race and ethnicity, education, and marital status combinations) for the 50 states and District of Columbia. This was done separately (for 72 sex, race and ethnicity, education, and marital status combinations) for persons aged ≥51 years. Results from the four logistic regressions were applied to the population compositions to estimate the proportion of HAAs aged 18–50 years (NHANES, NSFG, and GSS) and ≥51 years (GSS only) by stratum.

In the third stage, our weighted averages across surveys used a random-effects model; weights were the inverse of within and between survey variances [21]. Special considerations for meta-analysis to synthesize survey-based estimates are different sampling frames, subpopulations, question wording, and data collection timeframes [22]. To address these issues, we used a consistent “past 12 months” definition of heterosexual activity and limited the meta-analysis to ages 18–50 years because that range was common across surveys. Regarding timeframes, no adjustments were made as it is unlikely that heterosexual activity varied at the state level during the data coverage of the surveys used to estimate past-year heterosexual activity (2013–2018).

In the fourth stage, we tabulated numbers of HAAs separately for persons aged 18–50 years (meta-analysis results from NHANES, NSFG, and GSS) and persons ≥51 years (GSS only).

Standard errors of statewide probabilities of heterosexual activity are cumulative from the different methods in each stage. In the first stage, national estimates with standard errors were bootstrapped to provide standard errors for state estimates. In the third stage, random-effects models synthesized standard errors from survey-based estimates among respondents aged 18–50 years. The standard error for all adults was based on the sum of variances of synthesized results for adults aged 18–50 and ≥51 years, assuming the two estimates were independent.

All logistic regression analyses were done in SAS 9.4 (proc survey logistic) [23]. The marandom SAS macro was used for meta-analysis [24].

### Data sources and methods for HIV diagnoses

HIV diagnoses in 2018 were obtained from the Centers for Disease Control and Prevention’s AtlasPlus [25]. We included all persons aged ≥13 years reporting heterosexually acquired HIV. This risk reporting is consistent with our selection of the term HAA, rather than heterosexuals, because sexual orientation is fluid. The classification of HIV risk factor at the time of diagnosis is specific to the mode of transmission presumed to be related to an individual’s HIV acquisition and not sexual orientation. We included persons in the “Black/African American,” “Hispanic/Latino,” and “White” race and ethnicity categories; we excluded “multiple races” because there was no corresponding denominator available to produce HAA estimates. Our focus on the three racial and ethnic groups resulted in <5% of diagnoses being removed from the analysis (2.2% of male and female heterosexually-acquired diagnoses were in the combined categories of “American Indian/Alaska Native,” “Asian,” and “Native Hawaiian/Other Pacific Islander”; and another 2.2% of diagnoses were in the “Multiple Races” category).

We included all 50 states and the District of Columbia, although AtlasPlus did not contain HIV diagnoses for New Hampshire and diagnoses were not reported by transmission category and race and ethnicity for seven additional states (Delaware, Idaho, Massachusetts, Missouri, Montana, North Dakota, and Vermont).

### Rates and disparity measures

We divided new diagnoses by estimated population size to estimate HIV rates per 100,000 HAAs nationally and by state for non-Hispanic White, non-Hispanic Black, and Hispanic adults (hereafter, White, Black, and Hispanic). As disparity measures, we calculated RDs and RRs comparing Black-to-White HAAs and Hispanic-to-White HAAs. All rates and disparity measures were estimated by sex. Hex maps of disparity measures were created using R version 3.6.2 [26]. We opted to use hex maps rather than a conventional map format because interpretations of maps can be biased if the size of the geographical boundary is not proportional to the size of the concept of interest (e.g., some states in the Mountain and West North Central divisions have large geographic areas but fewer diagnoses, whereas New England states and the District of Columbia are populous but have smaller land areas). The background map used publicly available hexagon boundaries for US states [27], and figures were produced using the ggplot2 package [28].

Although we present the standard errors for the estimated number of HAA men and women in each racial and ethnic group by state, we do not report a confidence interval for the diagnosis rates, RDs, and RRs because the standard errors for the denominators is small and the number of diagnoses reflect the full population and not a sample. The hex maps indicate a “not calculated” shading for the states where there were no diagnoses among White HAA men or women and a RR could not be estimated with a denominator of zero.

### Human subjects review

We relied on data that were all publicly available for direct download online in aggregate and anonymized form without use restrictions. The four surveys used (ACS, GSS, NFSG, and NHANES) went through their own human subjects review for the primary data collect, with details available on their documentation websites. HIV diagnosis data from AtlasPlus are reported in aggregate counts with suppressed values for small counts to reduce reidentification risk in accordance with Centers for Disease Control and Prevention guidelines. Because all data were fully anonymized, available for free public use, and at a granularity that made it impossible to reidentify respondents, we did not need to seek review from our human subjects committee.

## Results

### HIV rates and disparity measures among heterosexually active men

**Fig 2:**
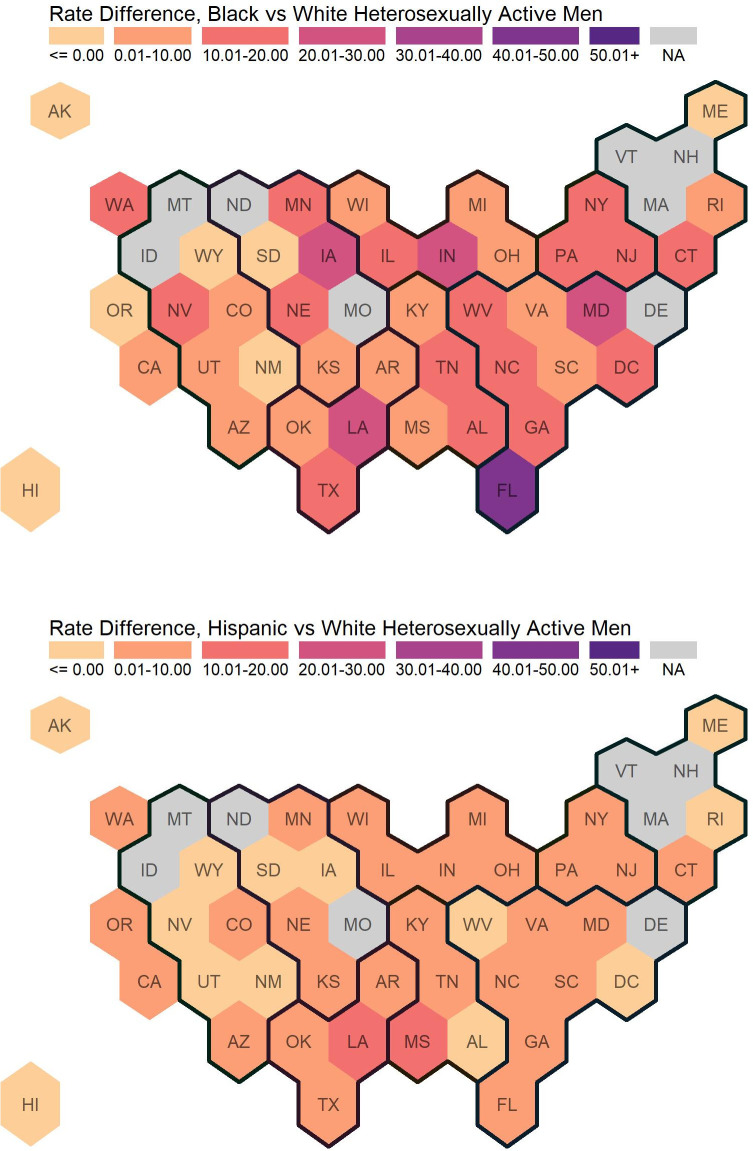
Rate differences of newly diagnosed HIV cases among heterosexually active Men Ages 18+. Regions are indicated with the dark black lines. Eight states did not report stratified data (by sex, transmission category, and race/ethnicity), and are indicated with “not available” (NA).

**Fig 3:**
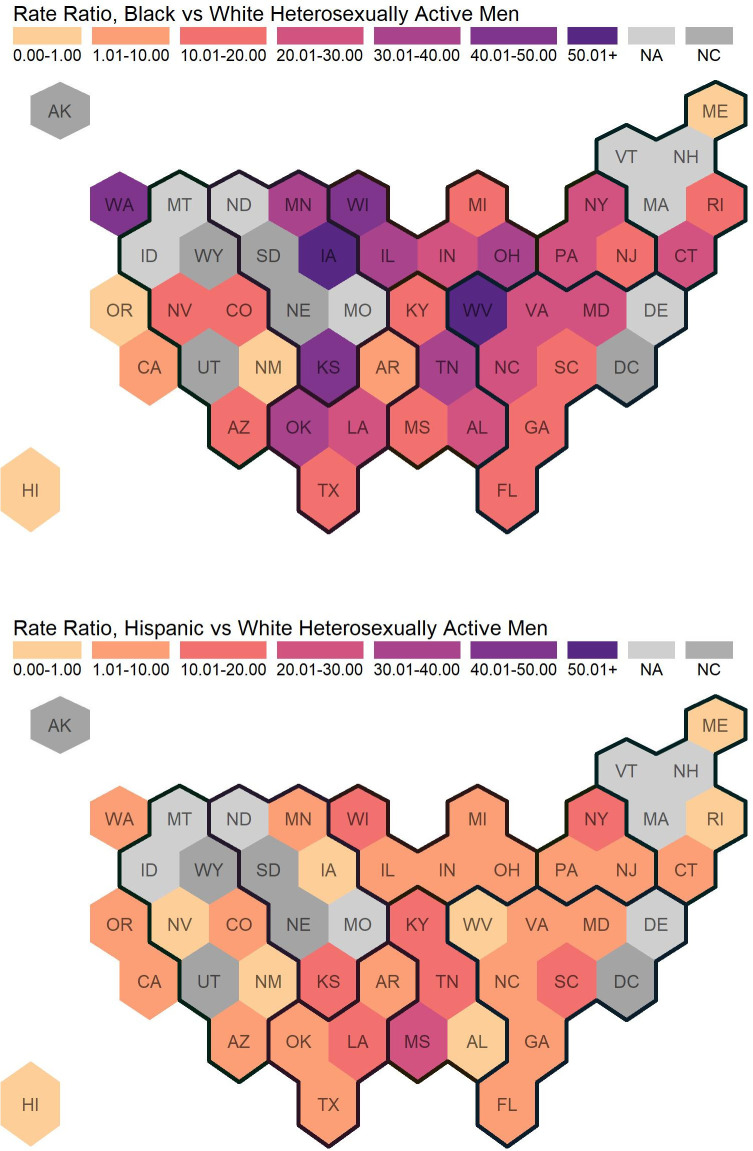
Rate ratios of newly diagnosed HIV cases among heterosexually active Men Ages 18+. Regions are indicated with the dark black lines. For most states in the lowest category with rate ratio between 0.00 and 1.00, there were no or very few new diagnoses among Black or Hispanic heterosexually active men. This commonly occurred in states with smaller populations. Eight states did not report stratified data (by sex, transmission category, and race/ethnicity), and are indicated with “not available” (NA). Six states do not have calculated rate ratios because there were 0 diagnoses among White heterosexually active men, and are indicated with “not calculated” (NC).

Table 1 displays estimated HIV diagnoses per 100,000 HAA White, Black, and Hispanic men; and accompanying RDs and RRs. For states without publicly available HIV diagnoses by race and ethnicity, we provide estimated HAAs only (“population size”). Fig 2 illustrates Black-to-White and Hispanic-to-White RDs and Fig 3 illustrates Black-to-White and Hispanic-to-White RRs.

**Table 1:** State-level estimates of heterosexually active Men Ages 18+, HIV diagnosis rates, and disparity measures.

	White		Black				Hispanic			
	Population size N (SE)	HIV rate per 100,000	Population size N (SE)	HIV rate per 100,000	Absolute Rate Difference	Relative Rate Ratio	Population size N (SE)	HIV rate per 100,000	Absolute Rate Difference	Relative Rate Ratio
**United States**	59,404,358 (315,167)	0.76	10,737,881 (95,957)	16.16	15.40	21.28	15,689,593 (271,982)	3.55	2.79	4.68
**West**										
*Mountain*										
Arizona	1,173,884 (12,520)	0.85	86,618 (945)	9.24	8.38	10.84	561,464 (13,769)	1.07	0.22	1.25
Colorado	1,174,051 (11,135)	0.34	68,929 (739)	4.35	4.01	12.77	319,903 (7,131)	1.88	1.53	5.51
Idaho	406,092 (3,514)	*	3,807 (53)	*	*	*	54,069 (1,286)	*	*	*
Montana	274,094 (2,748)	*	2,339 (29)	*	*	*	9,542 (217)	*	*	*
Nevada	459,478 (5,471)	1.09	71,555 (982)	16.77	15.68	15.41	225,483 (5,382)	0.89	-0.20	0.82
New Mexico	246,771 (2,715)	0.41	13,999 (174)	0.00	-0.41	0.00	276,511 (6,918)	0.36	-0.04	0.89
Utah	683,868 (5,137)	0.00	10,466 (105)	9.55	9.55	*	109,592 (2,543)	0.00	0.00	*
Wyoming	149,688 (1,384)	0.00	1,891 (30)	0.00	0.00	*	15,939 (383)	0.00	0.00	*
*Pacific*										
Alaska	147,723 (1,292)	0.00	8,521 (87)	0.00	0.00	*	14,156 (274)	0.00	0.00	*
California	4,618,605 (49,980)	0.74	641,126 (7,724)	6.08	5.35	8.26	4,158,360 (95,713)	1.30	0.56	1.76
Hawaii	113,768 (1,074)	0.88	10,743 (89)	0.00	-0.88	0.00	37,601 (768)	0.00	-0.88	0.00
Oregon	943,288 (9,461)	0.32	23,559 (258)	0.00	-0.32	0.00	139,530 (3,179)	1.43	1.12	4.51
Washington	1,560,670 (14,279)	0.26	87,403 (981)	12.59	12.33	49.10	239,791 (5,242)	2.50	2.25	9.76
**Midwest**										
*East North Central*										
Illinois	2,371,136 (21,974)	0.34	464,443 (6,740)	12.27	11.94	36.38	596,370 (13,198)	2.35	2.01	6.96
Indiana	1,535,785 (15,011)	1.17	155,419 (2,068)	24.45	23.28	20.86	117,138 (2,614)	5.98	4.80	5.10
Michigan	2,242,639 (22,685)	0.36	357,031 (5,371)	4.48	4.12	12.56	127,176 (2,771)	0.79	0.43	2.20
Ohio	2,695,765 (26,038)	0.30	368,515 (5,454)	8.95	8.66	30.18	109,436 (2,426)	2.74	2.44	9.24
Wisconsin	1,424,127 (13,967)	0.14	85,884 (1,281)	6.99	6.85	49.75	97,240 (2,093)	2.06	1.92	14.65
*West North Central*										
Iowa	796,404 (7,611)	0.25	29,324 (335)	20.46	20.21	81.48	47,276 (1,108)	0.00	-0.25	0.00
Kansas	654,083 (5,772)	0.15	47,580 (567)	6.31	6.15	41.24	88,208 (2,037)	2.27	2.11	14.83
Minnesota	1,339,479 (12,579)	0.45	87,357 (912)	17.17	16.72	38.33	74,655 (1,583)	1.34	0.89	2.99
Missouri	1,424,054 (13,668)	*	179,958 (2,482)	*	*	*	65,965 (1,341)	*	*	*
Nebraska	449,056 (4,045)	0.00	23,795 (274)	12.61	12.61	*	51,942 (1,137)	3.85	3.85	*
North Dakota	198,341 (1,796)	*	7,103 (97)	*	*	*	7,433 (167)	*	*	*
South Dakota	212,521 (1,986)	0.00	5,081 (53)	0.00	0.00	*	8,403 (190)	0.00	0.00	*
**Northeast**										
*Mid-Atlantic*										
New Jersey	1,484,671 (13,771)	1.08	307,914 (4,019)	16.24	15.16	15.07	492,824 (10,736)	8.93	7.85	8.28
New York	3,269,051 (31,390)	0.46	767,367 (10,130)	13.29	12.83	28.97	1,010,127 (23,244)	5.35	4.89	11.65
Pennsylvania	2,932,223 (29,026)	0.78	353,146 (4,901)	16.14	15.36	20.58	227,514 (5,870)	7.03	6.25	8.97
*New England*										
Connecticut	727,733 (7,159)	0.69	97,808 (1,191)	17.38	16.69	25.30	147,432 (3,508)	4.75	4.06	6.91
Maine	370,904 (3,905)	0.27	5,336 (56)	0.00	-0.27	0.00	5,687 (95)	0.00	-0.27	0.00
Massachusetts	1,466,857 (13,811)	*	132,953 (1,522)	*	*	*	203,712 (4,857)	*	*	*
New Hampshire	367,685 (3,930)	*	5,788 (61)	*	*	*	12,961 (238)	*	*	*
Rhode Island	229,204 (2,427)	0.44	17,193 (227)	5.82	5.38	13.33	42,354 (993)	0.00	-0.44	0.00
Vermont	174,239 (1,771)	*	2,415 (30)	*	*	*	3,062 (53)	*	*	*
**South**										
*East South Central*										
Alabama	953,319 (8,914)	0.42	339,932 (5,096)	11.77	11.35	28.04	54,467 (1,113)	0.00	-0.42	0.00
Kentucky	1,092,234 (10,548)	0.27	97,383 (1,391)	4.11	3.83	14.95	43,250 (930)	4.62	4.35	16.84
Mississippi	499,487 (4,731)	0.60	288,912 (4,133)	9.35	8.74	15.56	24,256 (503)	12.37	11.77	20.59
Tennessee	1,461,672 (13,198)	0.41	293,727 (4,113)	13.62	13.21	33.18	94,183 (2,035)	5.31	4.90	12.93
*South Atlantic*										
Delaware	178,563 (1,875)	*	55,067 (713)	*	*	*	22,955 (519)	*	*	*
District of Columbia	81,695 (1,093)	0.00	82,525 (1,373)	19.39	19.39	*	21,390 (372)	0.00	0.00	*
Florida	3,425,168 (38,154)	3.18	854,686 (11,262)	48.67	45.49	15.29	1,531,743 (33,161)	10.38	7.20	3.26
Georgia	1,642,325 (14,489)	1.46	837,008 (10,939)	19.95	18.49	13.65	261,482 (5,406)	5.74	4.28	3.93
Maryland	935,946 (8,452)	0.85	485,774 (6,149)	21.41	20.55	25.05	165,035 (3,156)	8.48	7.63	9.92
North Carolina	1,955,965 (17,169)	0.46	583,899 (7,983)	11.30	10.84	24.57	247,417 (5,244)	2.43	1.96	5.27
South Carolina	954,171 (9,062)	0.84	353,827 (5,158)	9.89	9.05	11.80	77,594 (1,624)	9.02	8.18	10.76
Virginia	1,598,374 (12,882)	0.44	444,463 (5,541)	10.12	9.69	23.12	219,671 (3,928)	2.28	1.84	5.20
West Virginia	495,241 (5,534)	0.20	20,121 (298)	14.91	14.71	73.84	6,748 (120)	0.00	-0.20	0.00
*West South Central*										
Arkansas	642,264 (6,013)	1.25	118,110 (1,744)	7.62	6.37	6.12	57,684 (1,417)	3.47	2.22	2.78
Louisiana	803,395 (7,892)	1.24	377,785 (5,870)	26.47	25.23	21.27	70,075 (1,429)	14.27	13.03	11.46
Oklahoma	772,837 (7,060)	0.26	77,017 (972)	9.09	8.83	35.12	105,034 (2,631)	1.90	1.65	7.36
Texas	3,594,194 (31,825)	1.09	894,670 (10,721)	12.85	11.77	11.85	2,925,051 (67,786)	2.87	1.79	2.65

***Abbreviations***: not available (NA), not calculated (NC).

***Notes***: Population refers to the number of men aged 18 and over reporting exclusively heterosexual activity in the past 12 months. Men who report having sex with both men and women are excluded. Populations are estimates and derived from a model-based synthesis of the National Health and Nutrition Examination Survey, National Survey of Family Growth, General Social Survey, and American Community Survey. Rates use the adjusted denominators of heterosexually active men. Rate ratios comparing demographics groups are calculated as the rate among Black and Hispanic heterosexually active men divided by the rate among White heterosexually active men and rate differences are calculated as the absolute difference between groups. Rate ratios of 1.0 and rate differences of 0.0 would indicate no disparities. Rates and rate ratios of 0.0 are for categories where there were no diagnoses among non-White persons in 2018. This may occur when there are few total new diagnoses in the state. Eight states did not report stratified data (by sex, transmission category, and race/ethnicity), and are indicated with “NA.” Six states do not have calculated rate ratios because there were 0 diagnoses among White heterosexually active men, and are indicated with “NC.”.

Nationally, the HIV diagnosis rate among states reporting data and the District of Columbia was 3.09 per 100,000 HAA men (for all race and ethnicity categories including “other races”). The HIV diagnosis rates stratified by race and ethnicity were 0.76, 16.16, and 3.55 per 100,000 HAA White, Black, and Hispanic men, respectively. Black-to-White and Hispanic-to-White RDs were 15.40 and 2.79, respectively. Black-to-White and Hispanic-to-White RRs were 21.28 and 4.68, respectively. Across states and the District of Columbia, HIV diagnosis rates among HAA men ranged from 0.00 to 3.18 among 100,000 White HAAs (median: 0.42), from 0.00 to 48.67 per 100,000 Black HAAs (median: 10.12), and from 0.00 to 14.27 100,000 Hispanic HAAs (median: 2.27). Black-to-White and Hispanic-to-White RDs among HAA men ranged from -0.88 to 45.49 (median: 9.69) and from -0.88 to 13.03 (median: 1.84), respectively. Black-to-White and Hispanic-to-White RRs among HAA men ranged from 0 to 81.48 (median: 20.58) and from 0 to 20.59 (median: 5.20), respectively.

Comparing census regions, HIV diagnosis rates among White HAA men were highest in the South (1.15 per 100,000 HAA men), followed by the Northeast (0.68 per 100,000 HAA men), West (0.55 per 100,000 HAA men), and Midwest (0.39 per 100,000 HAA men). Similar to White men, Black men had the highest HIV diagnosis rates in the South (19.42 per 100,000 HAA men); however, the rank ordering of the three other regions differed between Black and White men (Black men, Northeast: 14.66 per 100,000 HAA men; Midwest: 10.90 per 100,000 HAA men; West: 7.22 per 100,000 HAA men). There were different regional patterns among Hispanic HAA; HIV diagnosis rates were highest in the Northeast (6.28 per 100,000 HAA men), followed by the South (5.32 per 100,000 HAA men), Midwest (2.43 per 100,000 HAA men), and West (1.26 per 100,000 HAA men).

Although overall HIV diagnosis rates were highest in the South region, Black-to-White disparities were highest in the Midwest on the relative measure (RR, Midwest: 28.21, Northeast: 21.66, South: 16.84, West: 13.13) and Hispanic-to-White disparities were highest in the Northeast on the relative disparity measure (RR, Midwest: 6.29, Northeast: 9.28, South: 4.61, West: 2.30). Using the absolute disparity measure, the Black-to-White disparities were highest in the South which differed from findings from the relative measure (RD, Midwest: 10.51, Northeast: 13.98, South: 18.26, West: 6.67). Consistent with findings from the relative measure, the Hispanic-to-White disparities remained higher in the Northeast on the absolute measure (RD, Midwest: 2.04, Northeast: 5.61, South: 4.16, West: 0.71).

The hex map visualizations (Figs 2 and 3) support key findings from Table 1: among HAA men, Black-to-White disparities are higher than Hispanic-to-White disparities, and while RDs and RRs vary across states, disparities are ubiquitous across states without a clear geographical pattern. Some states including the District of Columbia have reverse disparities (RDs < 0.00 and RRs between 0.00 to 1.00); most of these states have small populations and their estimated rates are unstable.

### HIV rates and disparity measures among heterosexually active women

**Fig 4:**
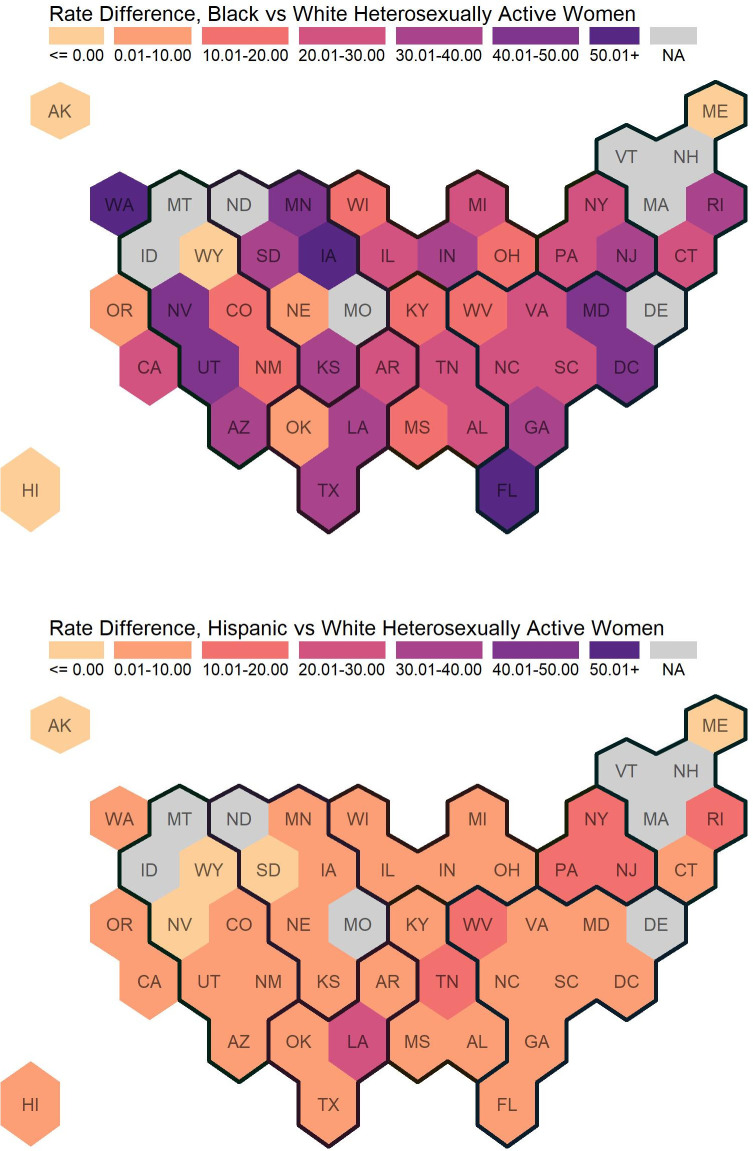
Rate differences of newly diagnosed HIV cases among heterosexually active Women Ages 18+. Regions are indicated with the dark black lines. Eight states did not report stratified data (by sex, transmission category, and race/ethnicity), and are indicated with “not available” (NA).

**Fig 5:**
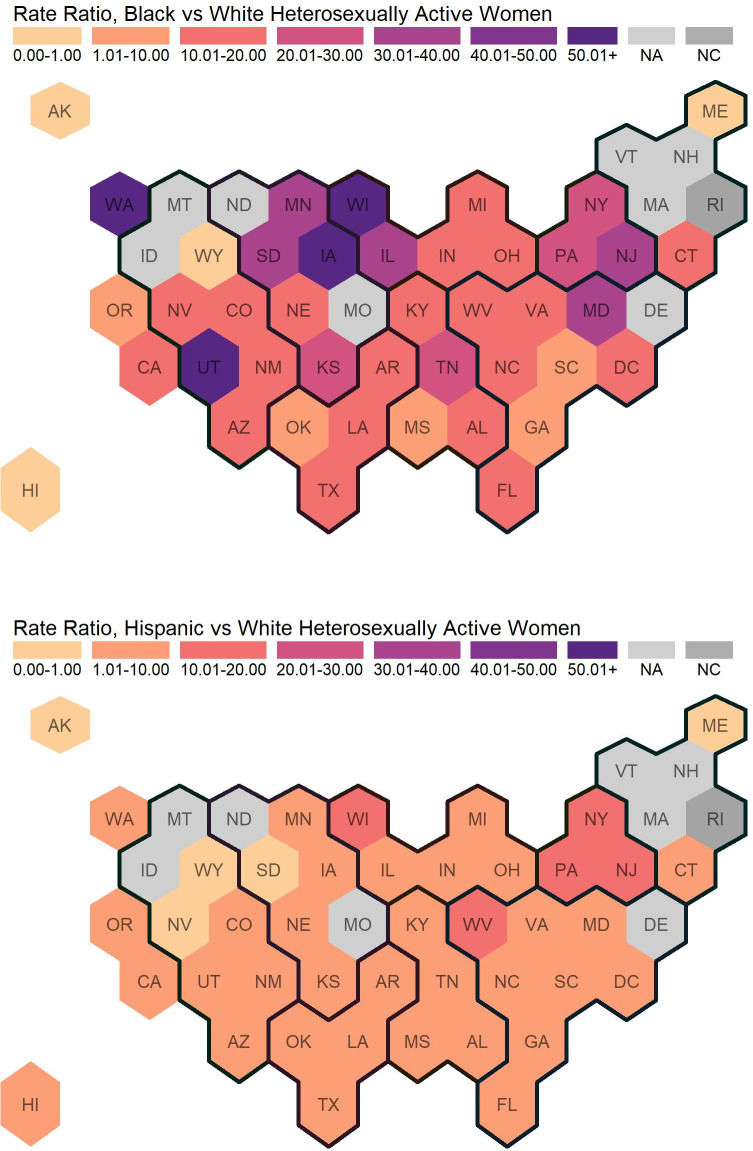
Rate ratios of newly diagnosed HIV cases among heterosexually active Women Ages 18+. Regions are indicated with the dark black lines. For most states in the lowest category with rate ratio between 0.00 and 1.00, there were no or very few new diagnoses among Black or Hispanic heterosexually active women. This commonly occurred in states with smaller populations. Eight states did not report stratified data (by sex, transmission category,, and race/ethnicity), and are indicated with “not available” (NA). One state does not have calculated rate ratios because there were 0 new diagnoses among White heterosexually active women, and are indicated with “not calculated” (NC).

Table 2, Figs 4 and 5 present similar information on HIV diagnoses, RDs, and RRs for HAA women by race and ethnicity.

**Table 2:** State-level estimates of heterosexually active Women Ages 18+, HIV diagnosis rates, and disparity measures.

	White		Black				Hispanic			
	Population size N (SE)	HIV rate per 100,000	Population size N (SE)	HIV rate per 100,000	Absolute Rate Difference	Relative Rate Ratio	Population size N (SE)	HIV rate per 100,000	Absolute Rate Difference	Relative Rate Ratio
**United States**	57,962,024 (645,200)	1.71	11,155,949 (50,264)	33.49	31.78	19.55	14,375,288 (204,695)	7.10	5.39	4.15
**West**										
*Mountain*										
Arizona	1,127,341 (16,218)	1.69	74,320 (1,120)	32.29	30.61	19.16	535,018 (14,720)	4.30	2.61	2.55
Colorado	1,126,784 (14,405)	1.06	55,200 (855)	18.12	17.05	17.01	293,208 (7,816)	2.39	1.32	2.24
Idaho	394,999 (5,174)	*	1,775 (24)	*	*	*	49,144 (1,291)	*	*	*
Montana	259,780 (3,347)	*	766 (14)	*	*	*	9,516 (273)	*	*	*
Nevada	413,948 (6,319)	3.62	67,662 (1,101)	45.82	42.19	12.64	205,639 (5,991)	2.43	-1.19	0.67
New Mexico	230,611 (3,309)	0.87	8,597 (161)	11.63	10.76	13.41	259,775 (8,548)	3.46	2.60	3.99
Utah	673,150 (7,371)	0.30	7,197 (90)	41.68	41.39	140.30	100,089 (2,234)	1.00	0.70	3.36
Wyoming	139,539 (1,774)	0.72	958 (15)	0.00	-0.72	0.00	13,526 (369)	0.00	-0.72	0.00
*Pacific*										
Alaska	124,276 (1,626)	0.80	5,410 (101)	0.00	-0.80	0.00	12,533 (200)	0.00	-0.80	0.00
California	4,297,972 (61,541)	1.98	598,708 (11,311)	24.39	22.41	12.33	3,821,434 (120,655)	4.55	2.58	2.30
Hawaii	87,707 (1,050)	2.28	5,431 (76)	0.00	-2.28	0.00	34,750 (656)	2.88	0.60	1.26
Oregon	920,514 (13,514)	1.41	18,362 (284)	10.89	9.48	7.71	124,444 (2,600)	4.02	2.61	2.85
Washington	1,491,131 (20,474)	0.74	65,560 (893)	108.30	107.56	146.81	212,067 (4,453)	5.19	4.45	7.03
**Midwest**										
*East North Central*										
Illinois	2,334,548 (31,333)	0.94	501,544 (9,084)	30.51	29.56	32.37	528,668 (16,049)	3.78	2.84	4.01
Indiana	1,523,333 (21,459)	1.71	165,057 (2,645)	32.11	30.40	18.81	102,647 (2,428)	5.85	4.14	3.42
Michigan	2,194,001 (30,353)	1.19	377,168 (6,937)	21.21	20.03	17.90	118,100 (2,682)	7.62	6.44	6.43
Ohio	2,667,200 (39,277)	1.31	381,390 (7,311)	18.62	17.30	14.19	98,608 (2,248)	3.04	1.73	2.32
Wisconsin	1,389,359 (18,154)	0.29	91,705 (1,471)	15.27	14.98	53.03	86,907 (1,966)	3.45	3.16	11.99
*West North Central*										
Iowa	784,128 (9,875)	0.89	24,548 (292)	52.96	52.06	59.32	40,659 (1,032)	7.38	6.49	8.27
Kansas	644,127 (8,064)	1.09	41,473 (665)	31.35	30.26	28.84	78,409 (2,011)	5.10	4.01	4.69
Minnesota	1,309,936 (15,934)	1.15	81,250 (1,019)	45.54	44.39	39.77	65,971 (1,208)	4.55	3.40	3.97
Missouri	1,407,660 (19,369)	*	193,717 (3,532)	*	*	*	58,900 (1,264)	*	*	*
Nebraska	440,807 (5,355)	0.68	22,140 (312)	9.03	8.35	13.27	45,432 (1,184)	2.20	1.52	3.23
North Dakota	184,050 (2,127)	*	4,320 (58)	*	*	*	5,946 (103)	*	*	*
South Dakota	203,216 (2,410)	0.98	3,218 (38)	31.08	30.09	31.57	6,000 (167)	0.00	-0.98	0.00
**Northeast**										
*Mid-Atlantic*										
New Jersey	1,461,140 (18,826)	0.96	325,471 (6,017)	35.03	34.07	36.56	446,742 (12,636)	11.42	10.46	11.91
New York	3,211,089 (46,056)	1.03	815,532 (14,709)	29.92	28.89	29.11	945,152 (29,316)	12.06	11.03	11.74
Pennsylvania	2,893,179 (41,172)	0.97	367,950 (6,945)	28.26	27.30	29.21	210,006 (5,007)	13.81	12.84	14.27
*New England*										
Connecticut	719,505 (9,650)	1.39	101,995 (1,669)	27.45	26.06	19.75	138,924 (3,337)	7.20	5.81	5.18
Maine	367,705 (5,281)	1.63	3,447 (33)	0.00	-1.63	0.00	5,522 (110)	0.00	-1.63	0.00
Massachusetts	1,478,839 (21,128)	*	133,200 (2,102)	*	*	*	195,289 (4,340)	*	*	*
New Hampshire	361,171 (5,016)	*	3,800 (57)	*	*	*	12,106 (236)	*	*	*
Rhode Island	228,965 (3,584)	0.00	16,069 (219)	31.12	31.12	*	38,326 (972)	15.66	15.66	*
Vermont	170,925 (2,421)	*	1,580 (22)	*	*	*	3,485 (80)	*	*	*
**South**										
*East South Central*										
Alabama	950,337 (12,645)	1.79	372,153 (6,196)	22.30	20.51	12.47	45,653 (1,035)	2.19	0.40	1.22
Kentucky	1,087,190 (15,693)	2.02	92,439 (1,688)	20.55	18.53	10.16	34,174 (626)	5.85	3.83	2.89
Mississippi	495,282 (6,454)	4.04	314,015 (5,702)	23.25	19.21	5.76	19,225 (364)	10.40	6.37	2.58
Tennessee	1,460,213 (20,339)	1.30	312,386 (5,270)	30.09	28.79	23.13	78,361 (1,675)	11.49	10.18	8.83
*South Atlantic*										
Delaware	179,758 (2,644)	*	58,702 (927)	*	*	*	20,427 (449)	*	*	*
District ofColumbia	81,689 (1,748)	3.67	90,157 (1,925)	53.24	49.57	14.50	18,694 (465)	10.70	7.03	2.91
Florida	3,289,658 (49,089)	3.53	887,940 (14,891)	60.48	56.95	17.15	1,422,010 (44,211)	12.45	8.92	3.53
Georgia	1,625,912 (20,737)	4.31	925,951 (14,037)	41.58	37.27	9.66	227,269 (4,824)	11.88	7.57	2.76
Maryland	922,801 (11,672)	1.19	522,066 (8,580)	41.95	40.76	35.19	143,122 (2,909)	7.69	6.49	6.45
North Carolina	1,944,272 (25,461)	2.01	629,406 (10,430)	22.40	20.40	11.17	214,343 (4,254)	7.00	4.99	3.49
South Carolina	950,097 (12,579)	3.16	382,068 (7,585)	28.79	25.63	9.12	63,388 (1,318)	9.47	6.31	3.00
Virginia	1,560,862 (19,543)	1.41	457,563 (7,989)	26.44	25.03	18.76	196,602 (3,844)	5.09	3.68	3.61
West Virginia	484,448 (7,577)	1.03	15,850 (314)	12.62	11.59	12.23	6,757 (180)	14.80	13.77	14.34
*West South Central*										
Arkansas	632,027 (8,818)	2.22	126,343 (2,203)	24.54	22.32	11.08	50,813 (1,223)	3.94	1.72	1.78
Louisiana	793,770 (11,483)	3.91	411,128 (7,448)	41.35	37.44	10.59	54,951 (1,490)	32.76	28.85	8.39
Oklahoma	757,753 (10,370)	1.98	74,708 (1,216)	10.71	8.73	5.41	91,194 (2,271)	8.77	6.79	4.43
Texas	3,485,390 (45,094)	2.35	925,534 (13,327)	36.95	34.60	15.71	2,704,844 (87,217)	7.65	5.30	3.25

***Abbreviations***: not available (NA), not calculated (NC).

***Notes***: Population refers to the number of women aged 18 and over reporting heterosexual activity in the past 12 months. For women, this includes both women who have sex with men exclusively, and women who have sex with both men and women. Populations are estimates and derived from a model-based synthesis of the National Health and Nutrition Examination Survey, National Survey of Family Growth, General Social Survey, and American Community Survey. Rates use the adjusted denominators of heterosexually active women. Rate ratios comparing demographics groups are calculated as the rate among Black and Hispanic heterosexually active women divided by the rate among White heterosexually active women and rate differences are calculated as the absolute difference between groups. Rate ratios of 1.0 and rate differences of 0.0 would indicate no disparities. Rates and rate ratios of 0.0 are for categories where there were no diagnoses among non-White persons in 2018. This may occur when there are few total new diagnoses in the state. Eight states did not report stratified data (by sex, transmission category, and race/ethnicity), and are indicated with “NA.” One state does not have calculated rate ratios because there were 0 diagnoses among White women, and is indicated with “NC.”.

Nationally, the HIV diagnosis rate among states reporting data and the District of Columbia was 6.57 per 100,000 HAA women (for all race and ethnicity categories including “other”). The HIV diagnosis rates stratified by race and ethnicity were 1.71, 33.49, and 7.10 per 100,000 White, Black, and Hispanic HAA women, respectively. Black-to-White and Hispanic-to-White RDs were 31.78 and 5.39, respectively. Black-to-White and Hispanic-to-White RRs were 19.55 and 4.15, respectively. Across states and the District of Columbia, HIV diagnosis rates among HAA women ranged from 0.00 to 4.30 per 100,000 White HAA women (median: 1.31), from 0.00 to 108.30 per 100,000 Black HAA women (median: 28.26), and from 0.00 to 32.76 per 100,000 Hispanic HAA women (median: 5.19). Black-to-White and Hispanic-to-White RDs among HAA women ranged from -2.28 to 107.56 (median: 26.06) and from -1.63 to 28.85 (median: 4.02), respectively. Black-to-White and Hispanic-to-White RRs among HAA women ranged from 0 to 146.81 (median: 15.10) and from 0 to 14.34 (median: 3.39), respectively.

HIV diagnosis rates among White HAA women were highest in the South (2.51 per 100,000 HAAs), followed by the West, Midwest, and Northeast regions (1.53, 1.09, and 1.02 per 100,000 HAAs, respectively). Among Black women, HIV diagnosis rates were highest in the South (36.44 per 100,000 HAAs) followed by the West, Northeast, and Midwest regions (31.74, 30.36, and 25.87 per 100,000 HAA women, respectively). Regional patterns differed for Hispanic women, who had the highest rates in the Northeast (11.77 per 100,000 HAAs) followed by the South, Midwest, and West (9.72, 4.44, and 4.20 per 100,000 HAAs, respectively). Although HIV diagnosis rates among Black and White HAA women were highest in the South region, Black-to-White and Hispanic-to-White disparities were highest in the Northeast on the relative disparity measure (Black-to-White RR, Midwest: 23.74, Northeast: 29.63, South: 14.49, West: 20.70; Hispanic-to-White RR, Midwest: 4.07, Northeast: 11.48, South: 3.69, West: 2.47). On the absolute disparity measure, the Black-to-White disparities were highest in the South which differed from findings from the relative measure (RD, Midwest: 24.78, Northeast: 29.33, South, 33.92, West: 30.21). Consistent with findings from the relative measure, the Hispanic-to-White disparities remained higher in the Northeast on the absolute measure (RD, Midwest: 3.35, Northeast: 10.74, South, 6.76, West: 2.67) The hex maps (Fig 3) illustrate these findings visually. The high Black-to-White and Hispanic-to-White rate ratios in the Northeast are likely driven by the Mid-Atlantic states, as three of the six New England states had insufficient data to calculate rate ratios.

### Comparison of rates and disparity measures with different denominators

Qualitatively, conclusions were similar when comparing HIV diagnosis rates and disparity measures between estimates using the Census population (irrespective of reported past-year sexual activity) versus our adjusted estimates among HAAs. HIV diagnosis rates and RDs had higher magnitudes when using estimated HAA population size as denominators, as HAAs are a subset of the US population. However, states’ relative rankings on HIV diagnosis rates, RDs, and RRs were consistent when using HAAs or total population as denominators.

## Discussion

Our analysis yielded several key findings. Nationally, heterosexually active women have a two-fold higher HIV diagnosis rate than heterosexually active men. Compared to White HAAs, HIV diagnosis rates were over 20 times higher among Black HAAs and over 4 times higher among Hispanic HAAs for both men and women. To help illustrate the magnitude of these disparities, for the Black-to-White RRs, 31 of 37 states including the District of Columbia had RR≥10 among HAA men and 33 of 42 states including the District of Columbia had RR≥10 among HAA women (omitting states with missing values due to data availability or insufficient data). These disparities were without notable regional patterns and suggests a system-wide failure particularly with respect to preventing HIV among Black and Hispanic women.

There are several advantages to our approach to estimating numbers of HAAs for use as denominators for heterosexually-acquired HIV. First, it likely yields more appropriate rates compared to using the full Census population. Although RRs as a measure of disparity were similar if rates were calculated using estimated HAAs versus the Census population, differences in rates can influence the magnitude of RDs. Furthermore, using rates with adjusted denominators of estimated HAAs allows providers to convey risk more appropriately to patients and facilitates comparisons of HIV burden across populations. These enhancements are important for documenting progress of policy initiatives such as the federal “Ending the HIV Epidemic in the U.S.” (EHE) strategy, which is in an early stage of implementation with jurisdictions recently receiving their second year of funding at the time of this analysis. Second, our HAA estimates can also be adapted to other measures related to HIV prevention and the HIV care continuum such use of pre-exposure prophylaxis.

Our finding that racial and ethnic disparities in HIV rates are consistent across states and the District of Columbia suggests that coordinated federal responses such as the EHE may be beneficial to ensure that the needs of all communities are met with a goal of reducing disparities. Although there was interstate variation in HIV diagnosis rates and disparity measures, in several instances this is driven by small numbers of diagnoses when stratifying by state and race and ethnicity. For example, there were reverse disparities (RD<0.0 and RR<1.0) in Alaska, Wyoming, and Maine and disproportionately large Black-to-White disparities in Iowa; each of these states has small counts of new diagnoses. While substantial research highlights the Southeast as the leading edge of the US epidemic [29,30], we find that disparities persist across and within all regions. The federal EHE plan has the potential to address inequities, while considering different risk profiles and needs at the community level, because it provides targeted funding to 57 priority EHE areas that collectively comprise almost two-thirds of new HIV diagnoses among Black and Hispanic persons and funding recipients are required to develop plans to reduce disparities and allocate funding for those purposes [31].

There are several primary explanations for why the HIV diagnosis rate is twice as high among HAA women compared to men. First, HAA women may be more likely to be screened for HIV than HAA men in the context of receiving reproductive healthcare services. Second, there are physiological differences by sex, and receptive penile-vaginal intercourse has twice the likelihood of transmitting an infection per contact compared to insertive penile-vaginal intercourse [32]. Third, many infections among HAA women may be associated with male sex partners who are connected to MSM transmission networks [33]. Other reasons include lack of awareness of their male sexual partners’ HIV status and risk factors and higher engagement in risky behaviors among women who have been sexually abused [34], with women experiencing higher rates of intimate partner violence than men [35].

A robust literature describes how the legacy of historic racism and trauma to Black women from the era of slavery to modern times have contributed to worse sexual health outcomes among women; these include but are not limited to coerced medical experimentation, race-based events such as rape and lynching, inadequate healthcare, and social determinants of health [36–38]. This historical context highlights the importance of culturally responsive interventions to improve linkage to and retention in highly effective HIV prevention and high-quality HIV care for Black women. Our finding of higher HIV diagnosis rates among Black HAA women is consistent with other research findings on disparities in sexual health outcomes including Black women having higher rates of maternal morbidity and mortality [39], congenital syphilis among their newborns [40,41], higher rates of unplanned pregnancy among women with HIV [42], and lower rates of pre-exposure prophylaxis use than either men or White women [43]. More generally, Black populations including men experience disparities across health conditions including COVID-19 [44], diabetes [45], cancer [46], and others.

Our analysis has several limitations. Our state-level aggregation may mask local variation in HIV diagnosis rates that are attributable to higher likelihood of HIV acquisition in geographic areas with a higher background HIV prevalence. Due to data availability, our diagnoses include adolescents aged 13–17 years because the lowest age category in AtlasPlus is 13–24 years. However, adolescents comprise a small share of HIV infections nationally [6] and the likely impact on our estimated rates is minimal. State-level data by race and ethnicity were missing in AtlasPlus for eight states. Although we were able to calculate RDs for all states with available data, there were additional states where we were unable to calculate RRs because the denominator (diagnosis rates among White HAA males or females) was zero. In most instances except HAA men in DC, this phenomenon is likely because of the small number of diagnosed cases. This suggests that in general, disparity measures in states with small populations may be unstable and should be interpreted with caution. While outside the scope of our analysis, future work might explore methods to minimize the impact of small case counts when examining data with multiple stratifications (i.e., by state, sex, mode of transmission, race and ethnicity, and age group). Due to data availability and small numbers within certain strata, we were unable to examine other races and ethnicities including multiple races which collectively comprised <5% of all diagnoses nationally. Each survey used to estimate the number of HAAs has common limitations such as non-response bias and potential reporting bias for self-reported sexual activity. Due to data availability, the estimated number of HAAs aged ≥51 years is only based on GSS. In standardizing based on national data, we assume that the probability of heterosexual activity within each demographic strata is equivalent across states. While this assumption was inadequate for past work estimating denominators of MSM due to differences in urban versus rural areas [47], this is a lesser concern for HAAs for whom lower stigma. While we believe that our rates using estimated HAAs as denominators are likely more appropriate than rates using the total Census population, we did not do a formal validity analysis. There are known racial and ethnic disparities in receiving late HIV diagnoses [48–50], and our analysis of new diagnoses does not account for underdiagnosis among racial and ethnic minorities. Lastly, the higher observed rates among Black women may be partially a surveillance artifact. Black women who move to the US from countries with high HIV prevalence are more likely to be diagnosed late and enter care late [51,52]; these individuals are identified as new diagnoses in surveillance systems.

Achieving the end of HIV as an epidemic in the US will require focused efforts to lessen persistent racial and ethnic disparities in HIV prevention and treatment, including among HAAs. The persistent HIV-related disparities in some Black and Hispanic communities confirm the need for culturally responsive interventions that address the social and structural factors associated with the disparities. Updating measures of HIV diagnosis and disparities using estimated HAAs as denominators can enable providers to convey risk to patients, provide comparisons of HIV burden across populations, and improve monitoring of national and state policy initiatives to end the epidemic.

## Supporting information

S1 Table Survey questions used to determine recent heterosexual activity. (DOCX)

S2 Table State-level estimates of heterosexually active Men and Women Ages 18+ and HIV diagnosis rates. (DOCX)
