# Supplementary material for: Racial and ethnic disparities in HIV diagnoses among heterosexually active persons in the United States nationally and by state, 2018
Source: PLoS One. 2021 Sep 20;16(9):e0257583. doi: 10.1371/journal.pone.0257583 (PMC8451999; doi:10.1371/journal.pone.0257583)
Supplement: S1 Table — (DOCX) [file pone.0257583.s001.docx]

**S1 Table. Survey Questions Used to Determine Recent Heterosexual Activity**

| **Data Source** | **Relevant Survey Questions** |
| --- | --- |
| National Health and Nutrition Examination Survey (NHANES) | - In the past 12 months, with how many women have you had any kind of sex? (male respondents only) - In the past 12 months, with how many men have you had any kind of sex? (female respondents only) - In the past 12 months, with how many men have you had anal or oral sex? (male respondents only) |
| National Survey of Family Growth (NSFG) | - Thinking about the last 12 months, how many female sex partners have you had in the 12 months since (MO/YR FILL FOR 12 MOS BEFORE INTERVIEW)? Please count every partner, even those you had sex with only once in those 12 months. (male respondents only) - Thinking about the last 12 months, how many male sex partners have you had in the 12 months since (MO/YR FILL FOR 12 MOS BEFORE INTERVIEW)? Please count every partner, even those you had sex with only once in those 12 months. (male respondents only) - Thinking about the last 12 months, how many male sex partners have you had in the 12 months since (MO/YR FILL FOR 12 MOS BEFORE INTERVIEW)? Please count every partner, even those you had sex with only once in those 12 months. (female respondents only) |
| General Social Survey (GSS) | - How many sex partners have you had in the last 12 months? - Have your sex partners in the last 12 months been… Exclusively Male, Exclusively Female, both. |

Notes: NHANES only asks sexual behavior questions to respondents aged 18 to 59, NSFG is limited to respondents aged 15-49, and GSS asks all respondents aged 18 and older about their sexual activities.
