# Supplementary material for: Racial and ethnic disparities in HIV diagnoses among heterosexually active persons in the United States nationally and by state, 2018
Source: PLoS One. 2021 Sep 20;16(9):e0257583. doi: 10.1371/journal.pone.0257583 (PMC8451999; doi:10.1371/journal.pone.0257583)
Supplement: S2 Table — (DOCX) [file pone.0257583.s002.docx]

**S2 Table. State-level Estimates of Heterosexually Active Men and Women Ages 18+ and HIV Diagnosis Rates**

|  | **Men** | | | **Women** | | |
| --- | --- | --- | --- | --- | --- | --- |
|  | **Population size N(SE)** | | **HIV rate per 100,000** | **Population size N(SE)** | | **HIV rate per 100,000** |
| **United States** | 92,658,371 | (534,485) | 3.09 | 91,893,801 | (805,521) | 6.57 |
| Alabama | 1,387,263 | (11,119) | 3.39 | 1,415,997 | (14,486) | 7.63 |
| Alaska | 215,464 | (2,227) | 0.46 | 195,721 | (2,505) | 0.51 |
| Arizona | 1,970,287 | (21,858) | 1.27 | 1,924,846 | (25,211) | 3.79 |
| Arkansas | 850,030 | (6,850) | 2.35 | 845,983 | (9,406) | 5.56 |
| California | 11,216,493 | (127,932) | 1.27 | 10,962,744 | (156,875) | 4.07 |
| Colorado | 1,643,209 | (15,042) | 0.79 | 1,579,269 | (17,391) | 1.84 |
| Connecticut | 1,033,488 | (9,357) | 2.90 | 1,034,613 | (11,332) | 4.93 |
| Delaware | 272,041 | (2,370) | 5.88 | 276,960 | (2,918) | 7.22 |
| District of Columbia | 195,237 | (1,786) | 8.71 | 206,090 | (3,133) | 26.69 |
| Florida | 6,052,326 | (55,581) | 11.58 | 5,911,350 | (72,241) | 14.24 |
| Georgia | 2,895,890 | (20,492) | 7.25 | 2,967,485 | (26,700) | 16.78 |
| Hawaii | 397,082 | (7,923) | 0.25 | 408,596 | (8,261) | 1.47 |
| Idaho | 481,305 | (4,220) | 0.83 | 468,559 | (5,537) | 1.28 |
| Illinois | 3,669,856 | (30,326) | 2.23 | 3,655,226 | (39,584) | 5.55 |
| Indiana | 1,874,094 | (16,803) | 3.63 | 1,868,257 | (22,736) | 4.60 |
| Iowa | 905,509 | (8,107) | 0.88 | 887,751 | (10,365) | 2.93 |
| Kansas | 832,727 | (6,792) | 0.84 | 814,556 | (8,420) | 2.82 |
| Kentucky | 1,265,704 | (11,362) | 0.71 | 1,255,023 | (16,135) | 3.59 |
| Louisiana | 1,293,177 | (10,715) | 9.43 | 1,309,941 | (14,590) | 17.10 |
| Maine | 392,403 | (3,959) | 0.25 | 390,075 | (5,390) | 1.54 |
| Maryland | 1,721,723 | (12,200) | 7.96 | 1,757,691 | (15,558) | 14.17 |
| Massachusetts | 1,964,574 | (17,280) | 2.14 | 2,005,314 | (24,644) | 6.53 |
| Michigan | 2,865,670 | (25,744) | 0.94 | 2,854,669 | (32,666) | 4.13 |
| Minnesota | 1,600,875 | (13,226) | 1.44 | 1,575,621 | (17,318) | 3.55 |
| Mississippi | 828,636 | (6,949) | 3.98 | 848,338 | (9,099) | 11.43 |
| Missouri | 1,733,831 | (14,478) | 1.27 | 1,737,529 | (20,265) | 3.91 |
| Montana | 305,851 | (2,971) | 0.65 | 294,301 | (3,649) | 0.34 |
| Nebraska | 546,602 | (4,589) | 0.91 | 534,145 | (5,562) | 1.12 |
| Nevada | 844,720 | (9,188) | 2.37 | 806,975 | (10,224) | 6.57 |
| New Hampshire | 401,833 | (4,098) | * | 395,721 | (5,067) | * |
| New Jersey | 2,555,907 | (20,785) | 4.58 | 2,550,810 | (25,580) | 7.33 |
| New Mexico | 592,025 | (8,197) | 0.34 | 567,830 | (9,898) | 2.47 |
| New York | 5,591,516 | (48,744) | 3.43 | 5,648,286 | (66,216) | 7.49 |
| North Carolina | 2,931,714 | (21,880) | 2.87 | 2,963,381 | (29,267) | 6.82 |
| North Dakota | 226,045 | (1,942) | 2.21 | 210,445 | (2,304) | 6.18 |
| Ohio | 3,295,845 | (29,121) | 1.34 | 3,294,266 | (41,833) | 3.43 |
| Oklahoma | 1,095,688 | (9,123) | 1.00 | 1,092,466 | (12,417) | 3.39 |
| Oregon | 1,194,733 | (11,714) | 0.50 | 1,178,049 | (14,796) | 1.87 |
| Pennsylvania | 3,674,883 | (32,980) | 2.72 | 3,669,696 | (44,854) | 4.61 |
| Rhode Island | 304,862 | (3,110) | 0.98 | 303,900 | (4,218) | 3.95 |
| South Carolina | 1,425,222 | (11,388) | 3.65 | 1,445,096 | (16,083) | 10.59 |
| South Dakota | 244,940 | (2,378) | 0.41 | 235,806 | (2,831) | 1.27 |
| Tennessee | 1,905,393 | (15,423) | 2.68 | 1,923,090 | (21,617) | 6.60 |
| Texas | 7,887,208 | (83,003) | 3.11 | 7,683,774 | (98,445) | 8.63 |
| Utah | 848,430 | (5,928) | 0.24 | 834,094 | (7,651) | 0.72 |
| Vermont | 185,044 | (1,854) | 0.00 | 183,064 | (2,485) | 1.64 |
| Virginia | 2,459,429 | (16,196) | 2.44 | 2,460,636 | (22,863) | 6.42 |
| Washington | 2,146,412 | (17,920) | 1.02 | 2,092,069 | (24,051) | 4.83 |
| West Virginia | 532,128 | (5,643) | 0.75 | 519,234 | (7,719) | 1.73 |
| Wisconsin | 1,676,315 | (15,514) | 0.66 | 1,650,195 | (19,050) | 1.33 |
| Wyoming | 174,020 | (1,544) | 0.00 | 161,623 | (1,856) | 0.62 |

* Data are not available in AtlasPlus.

***Notes***: Population refers to the number of men and women aged 18 and over reporting exclusively heterosexual activity in the past 12 months. Men who report having sex with both men and women are excluded. For women, the population includes both women who have sex with men exclusively, and women who have sex with both men and women. Populations are estimates and derived from a model-based synthesis of the National Health and Nutrition Examination Survey, National Survey of Family Growth, General Social Survey, and American Community Survey. Rates use the adjusted denominators of heterosexually active adults.
